# The *Trypanosoma brucei* AIR9-like protein is cytoskeleton-associated and is required for nucleus positioning and accurate cleavage furrow placement

**DOI:** 10.1111/j.1365-2958.2012.08008.x

**Published:** 2012-03-05

**Authors:** Sophie F May, Lori Peacock, Cristina I C Almeida Costa, Wendy C Gibson, Laurence Tetley, Derrick R Robinson, Tansy C Hammarton

**Affiliations:** 1 Wellcome Trust Centre for Molecular Parasitology, Institute of Infection, Immunity and Inflammation, College of Medical, Veterinary and Life Sciences, University of GlasgowGlasgow G12 8TA, UK; 2 School of Clinical Veterinary Science, University of BristolLangford, Bristol BS40 7DU, UK; 3 School of Biological Sciences, University of BristolBristol BS8 1UG, UK; 4 Instituto de Higiene e Medicina Tropical, Universidade Nova de LisboaLisbon, Portugal; 5 School of Life Sciences, University of GlasgowGlasgow G12 8QQ, UK; 6 UMR-CNRS 5234, University of Bordeaux 233076 Bordeaux, France

## Abstract

AIR9 is a cytoskeleton-associated protein in *Arabidopsis thaliana* with roles in cytokinesis and cross wall maturation, and reported homologues in land plants and excavate protists, including trypanosomatids. We show that the *Trypanosoma brucei* AIR9-like protein, TbAIR9, is also cytoskeleton-associated and colocalizes with the subpellicular microtubules. We find it to be expressed in all life cycle stages and show that it is essential for normal proliferation of trypanosomes *in vitro*. Depletion of TbAIR9 from procyclic trypanosomes resulted in increased cell length due to increased microtubule extension at the cell posterior. Additionally, the nucleus was re-positioned to a location posterior to the kinetoplast, leading to defects in cytokinesis and the generation of aberrant progeny. In contrast, in bloodstream trypanosomes, depletion of TbAIR9 had little effect on nucleus positioning, but resulted in aberrant cleavage furrow placement and the generation of non-equivalent daughter cells following cytokinesis. Our data provide insight into the control of nucleus positioning in this important pathogen and emphasize differences in the cytoskeleton and cell cycle control between two life cycle stages of the *T. brucei* parasite.

## Introduction

*Trypanosoma brucei* is a flagellated protozoan parasite transmitted by the tsetse fly which causes Human African Trypanosomiasis (HAT, more commonly known as sleeping sickness) and a related disease in animals, termed N'gana. The trypanosome cell has a vermiform shape that is conferred by a subpellicular microtubule cytoskeleton lying just beneath the plasma membrane (Gull, 1999). The cytoskeleton comprises a cage of parallel and equally spaced microtubules, arranged with their positive ends towards the cell posterior (Robinson *et al*., 1995), that are cross-linked to each other and to the plasma membrane by microtubule-associated proteins (MAPs) (Sant'Anna *et al*., 2005). In addition, four specialized microtubules run in the opposite orientation as part of the flagellum attachment zone, underlying the flagellar pocket (Gull, 1999). The cytoskeleton remains assembled throughout the cell cycle, with newly formed microtubules interdigitating between existing ones, resulting in a semi-conservative mode of inheritance (Sherwin and Gull, 1989).

The trypanosome cell cycle is intricately linked to the life cycle; both are highly complex, and there is extensive evidence for different cell cycle regulation operating in different stages (Hammarton, 2007). The parasite life cycle involves multiple differentiation steps that give rise sequentially to a number of different life cycle stages in its two hosts (Van den Abbeele *et al*., 1999). Only some life cycle stages are proliferative (long slender bloodstream form, and procyclic and epimastigote forms in the tsetse fly), while others are thought to be arrested in G_0_/G_1_[metacyclic and short stumpy bloodstream form (Matthews and Gull, 1994)] or G_2_[mesocyclic (Van den Abbeele *et al*., 1999; Sharma *et al*., 2008)] phases of the cell cycle. All life cycle stages contain a number of single copy organelles, including the nucleus, the mitochondrion (whose DNA is organized into a disc-shaped structure known as the kinetoplast), basal body/flagellum complex and Golgi apparatus, which are replicated and segregated during the cell cycle in proliferative stages in a tightly orchestrated and precisely ordered process (Vaughan, 2010). Following organelle replication, a furrow ingresses unidirectionally from the anterior to posterior along the longitudinal axis of the cell, contrasting with the contractile actomyosin ring used by mammalian cells and yeasts to divide. Currently, in trypanosomes, it is not known mechanistically how the furrow divides and separates the cytoskeletal microtubules and plasma membrane, or what determines its path (Hammarton *et al*., 2007b), but differences in organelle positioning and cell shape of different life cycle stages suggest some aspects of cytokinesis will be life cycle stage specific. To date, a variety of molecular regulators of cytokinesis have been identified (Hammarton *et al*., 2005; 2007a,b; Li *et al*., 2008; 2009; 2010; Ma *et al*., 2010; Price *et al*., 2010), but the signal transduction pathways in which they operate have not yet been delineated. Several MAPs have also been shown to be important for cytokinesis, since their depletion [e.g. WCB (Baines and Gull, 2008) and CAP5.5/CAP5.5V (Olego-Fernandez *et al*., 2009)] or overexpression [e.g. CAP15 and CAP17 (Vedrenne *et al*., 2002)] results in abnormal cell cycle progression, increasing the proportion of post-mitotic cells and aberrant cell types such as zoids (anucleate cells with a single kinetoplast), multinucleate cells or cells undergoing aberrant furrowing, in the population. Additionally, a family of microtubule severing proteins are required for cytokinesis (Casanova *et al*., 2009; Benz *et al*., 2012).

AIR9 (Auxin-Induced in Root cultures 9) was first identified in *Arabidopsis thaliana* (AtAIR9) and reported to be an essential MAP with homologues in land plants and excavate protists, including *Trichomonas* and the trypanosomatids (Neuteboom *et al*., 1999; Buschmann *et al*., 2007). It possesses a disordered, basic, serine-rich microtubule-binding domain at its N-terminus followed by six leucine-rich repeats and eleven A9 domains, which are immunoglobulin-like domains commonly involved in mediating protein: protein or protein: ligand interactions. AtAIR9 displays a dynamic localization during the plant cell cycle. It localizes to cortical microtubules in interphase and labels the pre-prophase band (PPB), a transient microtubule array that marks the future cortical division site, at the end of G2 phase (Buschmann *et al*., 2006; Mueller *et al*., 2009). During cytokinesis, AtAIR9 labels the phragmoplast and, once the phragmoplast contacts the cell cortex, also labels the former site of the PPB at the cortical division site. It then outlines the new cross wall, where it is thought to play a role in the maturation of the cross wall (Buschmann *et al*., 2006). Thus, we hypothesized that the AIR9-like protein in *T. brucei* might also be important for cytokinesis. The *T. brucei* AIR9-like protein (TbAIR9) contains six leucine-rich repeats and five A9 domains, but lacks the N-terminal basic serine-rich domain (Buschmann *et al*., 2007), and hence it is not clear from its sequence whether it is able to interact with microtubules or the cytoskeleton or if it is a true orthologue of AtAIR9. In this study, we investigate the localization and function of TbAIR9 in procyclic and bloodstream *T. brucei*. We demonstrate that while TbAIR9, like AtAIR9, is able to associate with the parasite subpellicular cytoskeleton and plays a role in cytokinesis in bloodstream trypanosomes, it has a different role in procyclic trypanosomes, playing an important role in the spatial positioning of the nucleus. To our knowledge, this is the first protein involved in nucleus positioning to be identified in any kinetoplastid parasite. Our data also highlight key differences in cell cycle control between different life cycle stages of *T. brucei* and underscore the importance of the cytoskeleton and its associated proteins in cell division.

## Results

### *T. brucei* AIR9 is a cytoskeleton-associated protein

**Fig 1:**
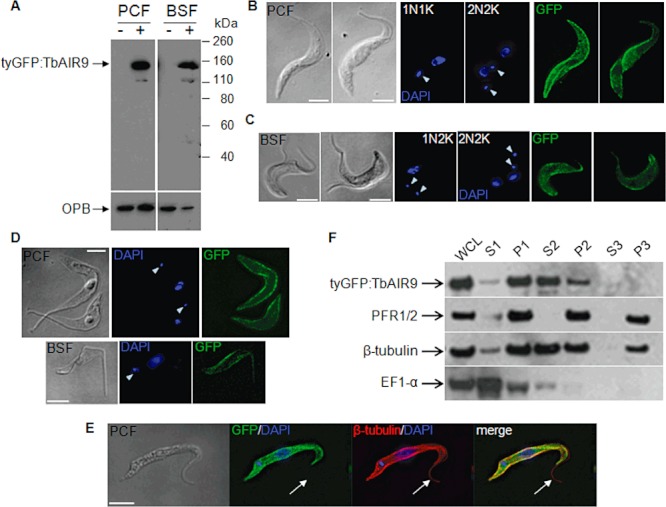
tyGFP:TbAIR9 is a cytoskeletal-associated protein but is not present in the flagellum. A. Western blot of lysates of procyclic (PCF) and bloodstream (BSF) *T. brucei* Lister 427 wild-type (−) and 427 pHG172 (tyGFP:TbAIR9; expected size = 139 kDa) (+) cells, probed with anti-GFP antibody (upper panels). The sizes of the molecular weight markers are indicated. Lower panels: same samples probed with anti-OPB antiserum as a loading control. B and C. Fluorescence microscopy images of tyGFP:TbAIR9-expressing procyclic (B) or bloodstream (C) stage cells. Panels from left to right: DIC images, DAPI staining (blue), tyGFP:TbAIR9 (green). The number of nuclei (N) and kinetoplasts (K) per cell are indicated, and arrowheads point to the kinetoplasts. D. Cytoskeletal preparations of procyclic (PCF, upper panels) and bloodstream (BSF, lower panels) *T. brucei*. Image panels as for (B and C). E. (Immuno)fluorescence analysis of procyclic whole cells. From left to right: DIC image; tyGFP:AIR9 (direct fluorescence; green)/DAPI (blue); anti-β-tubulin (KMX; red)/DAPI (blue); GFP/KMX/DAPI merge. Arrows indicate flagellum with a β-tubulin signal but not tyGFP:AIR9 fluorescence. Scale bars (B–E): 5 µm. F. Subcellular fractionation of procyclic cells expressing tyGFP:TbAIR9. Western blots of fractions [whole cell lysate (WCL), supernatants (S1-3) and pellets (P1-3), see *Experimental procedures* for full details] were probed with anti-TY, anti-PFR1/2, anti-β-tubulin and anti-EF1α antibodies, as indicated. A total of 10^6^ cell equivalents were loaded per lane.

To determine whether TbAIR9 localizes to the cytoskeleton in bloodstream and procyclic trypanosomes, cell lines that express TbAIR9 epitope-tagged at the N-terminus (tyGFP:TbAIR9) or at the C-terminus (TbAIR9:6ha) from the *TbAIR9* endogenous locus were generated and analysed by fluorescence microscopy (Figs 1 and S1). Expression of the fusion proteins was demonstrated by Western blotting with anti-GFP (Fig. 1A) or anti-HA antibodies (Fig. S1A). (Immuno)fluorescence microscopy of cell lines expressing tyGFP:TbAIR9 or TbAIR9:6ha revealed a brightly stained outline of the cell body in both life cycle stages (Fig. 1B and C, and Fig. S1B and C, respectively). TbAIR9 outlined the cell body throughout the cell cycle, and in cells undergoing mitosis or cytokinesis (cells with two nuclei and two kinetoplasts per cell (2N2K), as visualized by DAPI staining), the daughter cell body was also outlined. Identical patterns of TbAIR9 fluorescence were obtained for both fusion proteins in both life cycle stages, arguing that the observed localization was unlikely to be an artefact of the epitope tags. Further analysis of the tyGFP:TbAIR9 cell lines revealed that the tyGFP:TbAIR9 signal was retained in cytoskeleton preparations of both procyclic and bloodstream trypanosomes, indicating that it stably interacts with the cytoskeleton (Fig. 1D). Consistent with this, tyGFP:TbAIR9 colocalized with β-tubulin in the cell body of procyclic *T. brucei* cells, although the TbAIR9 signal did not extend into the flagellum (Fig. 1E). However, TbAIR9 was not observed to localize to the mitotic spindle, as revealed by immunofluorescence with the KMX anti-β-tubulin antibody (Birkett *et al*., 1985) (Fig. S2).

To confirm the localization of TbAIR9, subcellular fractionation of procyclic form cells expressing tagged TbAIR9 was performed, and fractions were Western blotted with antibodies against the relevant tags (Figs 1F and S1D). As controls, antibodies against the cytosolic protein, EF1α, the paraflagellar rod (PFR) proteins, PFR1 and PFR2, and β-tubulin were also employed to determine the success of the fractionation. EF1α was detected predominantly in the cytosolic fraction, while PFR signal was detected in the cytoskeletal and flagellar fractions, as expected (Kaur and Ruben, 1994; Kohl *et al*., 1999). β-Tubulin was present in the cytoskeletal and flagellar fractions (Kohl and Gull, 1998), although some β-tubulin was extracted from the cytoskeleton by the first salt wash. TbAIR9 displayed a similar distribution to β-tubulin although only a negligible amount of TbAIR9 remained associated with the flagellar fraction following the two salt extractions. A similar subcellular fractionation profile was obtained for bloodstream stage trypanosomes expressing TbAIR9:6ha (Fig. S1E). Thus, the subcellular fractionation and immunofluorescence data are consistent and suggest TbAIR9 is associated with the subpellicular cytoskeleton but not with the flagellum or mitotic spindle.

### RNAi-mediated downregulation of TbAIR9 in procyclic *T. brucei* preferentially depletes AIR9 from the cell posterior without grossly affecting cytoskeletal structure and slows population growth

**Fig 2:**
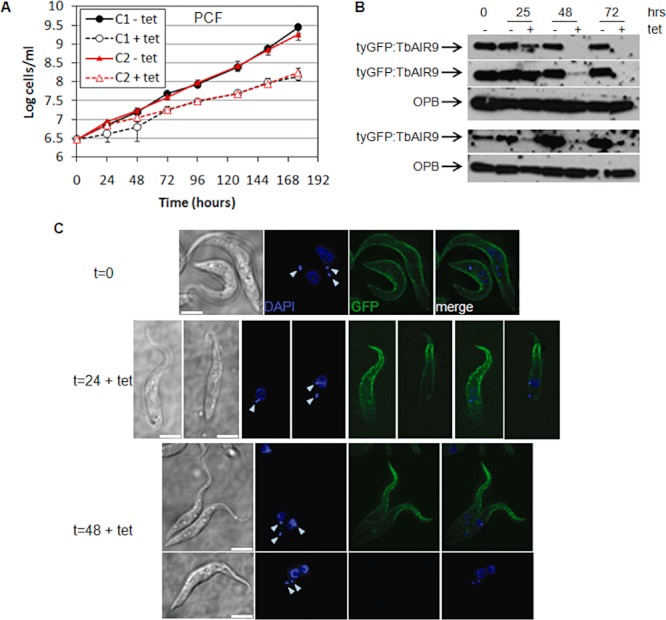
Depletion of TbAIR9 following induction of RNAi in procyclic trypanosomes occurs preferentially from the posterior end and reduces growth rate. A. Cumulative growth curves for two independent *TbAIR9* RNAi cell lines in the presence (+ tet) or absence (− tet) of tetracycline. B. Western blot of lysates of *TbAIR9* RNAi cell lines expressing tyGFP:TbAIR9 from one endogenous allele, probed with anti-GFP antibody or with anti-OPB antiserum as a loading control. Upper panels: clone 1; lower panels: clone 2. Two tyGFP:TbAIR9 blots of different exposures are presented for clone 1 to show residual tyGFP:TbAIR9 at 48 h post induction. Samples of cells grown in the presence or absence of tetracycline were taken every 24 h for 72 h, as indicated. C. Fluorescence microscopy images of *TbAIR9* RNAi cell lines expressing tyGFP:TbAIR9 from one endogenous allele. Cells were prepared for imaging at the time points (in hours) indicated. Panels from left to right: DIC images, DAPI staining (blue), tyGFP:TbAIR9 (green), GFP/DAPI merge. Arrowheads point to kinetoplasts. Scale bars: 5 µm.

To investigate the function of TbAIR9 in procyclic *T. brucei*, two independent clonal RNAi cell lines were generated. Following the induction of *TbAIR9* RNAi by the addition of tetracycline to the culture medium, both cell lines proliferated more slowly from 48 h post induction (Fig. 2A), with population doubling rates of 30.2 and 32.4 h (for clones 1 and 2 respectively) following induction compared with 17.8 and 19.0 h (for clones 1 and 2 respectively) in the absence of induction. To confirm that TbAIR9 was depleted following RNAi induction, one allele of *TbAIR9* in each RNAi cell line was replaced with *tyGFP:TbAIR9* and the depletion of TbAIR9 was monitored by Western blotting with anti-GFP antibody. In both RNAi cell lines, a substantial depletion in tyGFP:TbAIR9 was detected by 24 h post induction, with tyGFP:TbAIR9 undetectable by 72 h post induction (Fig. 2B). When observed by fluorescence microscopy, tyGFP:TbAIR9 was found to be preferentially depleted from these cells at their posterior ends (Fig. 2C), as has been observed for other trypanosome cytoskeleton-associated proteins (CAPs) and MAPs (Baines and Gull, 2008; Olego-Fernandez *et al*., 2009), and by 48 h post induction, the majority of cells displayed only very weak or no tyGFP:TbAIR9 signal. However, unlike depletion of other CAPs or MAPs, no gross cytoskeletal distortions were observed by transmission electron microscopy (TEM) following depletion of TbAIR9 (Fig. S3). At 72 h post induction, sub-pellicular microtubules were observed to remain parallel and equally spaced, and to associate with the plasma membrane normally (*n* = 43 cells). Flagellar axonemes displayed a normal 9 + 2 microtubule structure (*n* = 16) and no major defects were observed in the PFR, basal bodies or four specialized microtubules underlying the flagellar pocket. Negatively stained whole mount cytoskeleton preparations were also examined by TEM, confirming that depletion of AIR9 did not induce any gross abnormalities in the subpellicular microtubules or flagella (Fig. S4).

### Depletion of TbAIR9 in procyclic *T. brucei* results in a nucleus positioning defect and aberrant cytokinesis

**Fig 3:**
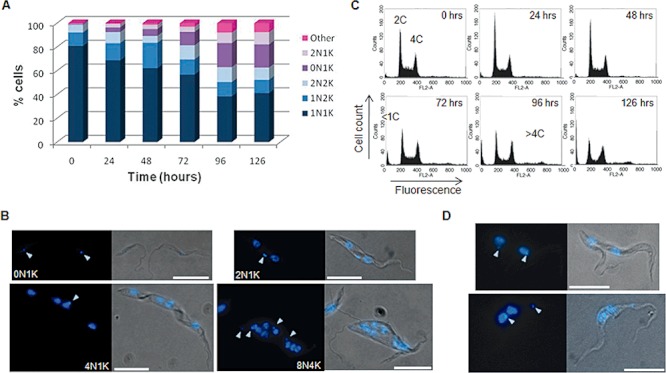
Depletion of TbAIR9 in procyclic *T. brucei* results in aberrant cell division. A. DAPI staining of *TbAIR9* RNAi cells following induction, at the time points indicated. The number of nuclei (N) and kinetoplasts (K) in > 200 cells/time point are indicated. ‘Other’ comprise mainly multinucleate cells. B. Images of DAPI-stained cells (left panels: DAPI; right panels: DAPI/DIC merge) with abnormal N/K configurations, as indicated. C. DNA content (flow cytometry) analysis of *TbAIR9* RNAi cells following induction, at the time points indicated. The ploidies of the peaks are indicated. D. DAPI stained images [as in (B)] of aberrantly dividing 2N2K cells. Arrowheads point to kinetoplasts. Scale bars: 10 µm. Data presented are for clone 1.

To investigate the reason for the growth defect, RNAi cell lines were analysed by DAPI staining and flow cytometry. Data obtained for the two independent clones were very similar, so only data for one clone is presented here. DAPI staining revealed the appearance of abnormal cell types from 24–48 h post induction, including 0N1K (zoids), 2N1K and, at later time points (> 96 h), cells with multiple nuclei and kinetoplasts (Fig. 3A and B). Flow cytometry of cells stained with propidium iodide revealed that cells with < 1C DNA content accumulated in the population over time, and at later time points, some cells with > 4C DNA content were also detected (Fig. 3C), consistent with the zoids and multinucleate cells, respectively, that were observed by DAPI staining. The zoids and 2N1K cells first appeared together in approximately equal numbers, suggesting that they could have arisen from the same progenitor cell. Indeed, 2N2K cells dividing unequally were observed in the population (Fig. 3D). At later time points however, the number of zoids exceeded the number of 2N1K cells, which can be accounted for by 2N1K cells undergoing additional rounds of organelle replication and being classified within the multinuclear/multikinetoplast cells, although occasionally, zoids were also observed budding off cells with multiple nuclei and kinetoplasts (data not shown).

**Fig 4:**
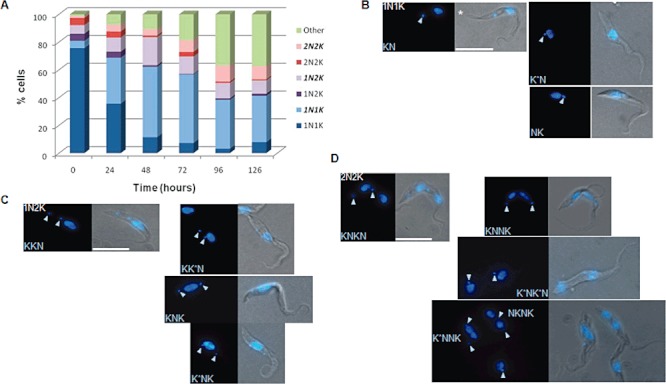
Depletion of TbAIR9 in procyclic trypanosomes results in aberrant positioning of the nucleus and kinetoplast. A. Prevalence of nucleus/kinetoplast (N/K) positioning defects following induction of *TbAIR9* RNAi. Data presented in Fig. 3A are reclassified according to whether cells displayed normal (standard font) or abnormal (bold italic font) N/K positioning. Data presented are for clone 1. B–D. DAPI stained images of 1N1K, 1N2K and 2N2K cell types, respectively, illustrating the range of N/K positioning phenotypes observed. Left panels: DAPI; right panels: DAPI/DIC merge. For reference, examples of cells displaying normal N/K positions (KN, KKN and KNKN, light blue font) for each cell type are presented in the top left of each figure panel. Organelles are listed in order from posterior to anterior. K^∧^: kinetoplast juxta-positioned to nucleus. Arrowheads point to kinetoplasts. Scale bars: 10 µm. Asterisk in (B) indicates posterior end of cell.

**Fig 5:**
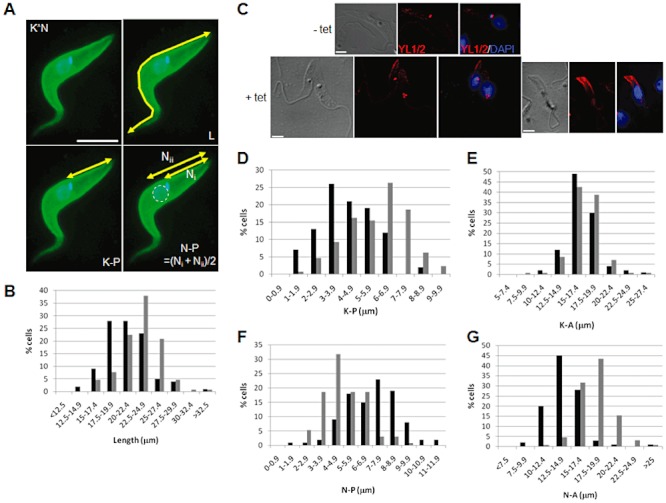
Morphometric analysis of procyclic *TbAIR9* RNAi cells. Cells were stained with anti-β-tubulin antibody (green) to outline the cytoskeleton and flagellum and co-stained with DAPI to visualize the nucleus (N) and kinetoplast (K). A. The following dimensions of the cell were measured in 1N1K cells: length of the cell (L); middle of the kinetoplast to posterior tip (K-P); centre of the nucleus to posterior tip (N-P), calculated as indicated. The kinetoplast to anterior tip (K-A) and nucleus to anterior tip (N-A) dimensions were calculated by subtracting K-P and N-P dimensions, respectively, from L. Measurements were binned as indicated and frequency distributions plotted for length (B), K-P (D), K-A (E), N-P (F) and N-A (G). Black bars: uninduced cells; grey bars: cells induced with tetracycline (tet) for 72 h. Data presented are for clone 1 [*n* = 100 (− tet) and 128 (+ tet)]. C. YL1/2 staining of procyclic *TbAIR9* RNAi cells, 72 h post induction with tetracycline (tet). Left panels: DIC images; middle panels: YL1/2 staining (red); right panels: YL1/2/DAPI merge. Staining of the posterior end and the basal bodies is visible. Scale bars: 3 µm.

Closer examination of the induced cells with normal nucleus and kinetoplast configurations revealed many cells with apparent defects in the positioning of these organelles that were apparent from 24 h post induction (Fig. 4A). Instead of the kinetoplast being located approximately midway between the nucleus and posterior pole in 1N1K cells, in many cases, the nucleus and kinetoplast were juxta-positioned, with the kinetoplast located to the lateral side of, or even anterior to, the nucleus (Fig. 4B). This phenotype became more severe and more prevalent over time. Similarly, 1N2K cells were observed with one or both kinetoplasts juxta-positioned or anterior to the nucleus (Fig. 4C), and a proportion of 2N2K cells, displayed a variety of N/K arrangements other than the normal (posterior to anterior) KNKN arrangement (Fig. 4D). The rapid onset of this phenotype following RNAi induction suggests that this defect is likely to be a direct effect of AIR9 depletion. However, the observed apparent defects in N/K positioning could have arisen from malpositioning of the nucleus or kinetoplast or both. Thus, the positions of the nucleus and kinetoplast in 1N1K cells that had been stained with a β-tubulin antibody to outline the cell and with DAPI to visualize DNA-containing organelles were examined. The distances from the posterior and anterior ends of the cell to the centres of the nucleus and kinetoplast in 1N1K cells displaying abnormal N/K positioning were measured and compared with those in 1N1K cells displaying normal N/K positioning in uninduced cell populations (Fig. 5A, Table 1). 1N1K cells from induced populations were found, on average, to be 10–15% (2.2–3.0 µm) longer than those from uninduced populations (Fig. 5B and data not shown; Table 1) (clone 1: *t*_227_ = 5.25, *P* < 0.0001; clone 2: *t*_245_ = 7.62, *P* < 0.0001). Since *T. brucei* cell length increases during differentiation from the bloodstream to procyclic form as well as during the cell cycle via active microtubule extension at the posterior end of the parasite (Sherwin *et al*., 1987; Matthews *et al*., 1995), *TbAIR9* RNAi 1N1K cells were analysed by immunofluorescence with the YL1/2 antibody, which recognizes tyrosinated α-tubulin and is a marker for actively extending microtubules (Kilmartin *et al*., 1982) to determine whether posterior end microtubule extension might account for their increased length. The posterior ends of induced *TbAIR9* RNAi cells stained brightly with YL1/2 compared with non-induced cells at 72 h post induction (Fig. 5C), indicating that microtubules were actively extending at the posterior ends of these cells.

**Table 1:** Morphometric measurements of 1N1K cells in procyclic *TbAIR9* RNAi cells 72 h post induction.

	Length (L)		Kinetoplast to posterior (K-P)		Kinetoplast to anterior (K-A)		Nucleus to posterior (N-P)		Nucleus to anterior (N-A)	
					
Clone	Mean (µm)	SD (µm)	Mean (µm)	SD (µm)	Mean (µm)	SD (µm)	Mean (µm)	SD (µm)	Mean (µm)	SD (µm)
C1: − tet	21.22	3.13	4.23	1.43	16.99	2.30	7.05	1.79	14.17	2.32
C1: + tet	23.48	3.31	5.91	1.65	17.57	2.55	5.01	1.39	18.47	2.59
C2: − tet	19.10	2.78	3.60	1.36	15.50	2.15	6.33	1.67	12.76	2.39
C2: + tet	22.04	3.13	5.39	1.62	16.64	2.40	4.60	1.36	17.43	2.91

The table shows the mean values and standard deviations (SD) of cellular dimensions of ≥ 100 1N1K cells from procyclic *TbAIR9* RNAi cells incubated in the presence or absence of tetracycline (tet) for 72 h.

In addition to the increased cell length, the kinetoplast to posterior end (K-P) distance increased on average by 1.7–1.8 µm (clone 1: *t*_227_ = 8.11, *P* < 0.0001; clone 2: *t*_245_ = 9.19, *P* < 0.0001), while the mean kinetoplast to anterior end (K-A) distance also increased, but to a lesser extent [0.6 µm (clone 1: *t*_227_ = 1.79, *p* = 0.075) or 1.1 µm (clone 2: *t*_245_ = 3.83, *P* < 0.001)] (Fig. 5D and E and data not shown; Table 1). Given the active microtubule extension at the posterior end of the parasite, the increase in K-P distance can be accounted for by cell elongation at the posterior pole rather than a movement of the kinetoplast itself towards the anterior end of the cell. Indeed, the slight increase in the K-A distance suggests that the kinetoplast may move slightly towards the posterior end following depletion of TbAIR9. In contrast to the kinetoplast distances, the average nucleus to posterior (N-P) distance decreased by 1.8–2.0 µm (clone 1: *t*_227_ = 9.68, *P* < 0.0001; clone 2: *t*_245_ = 8.92, *P* < 0.0001), while a more dramatic increase [4.3–4.7 µm (clone 1: *t*_227_ = 13.04, *P* < 0.0001; clone 2: *t*_245_ = 13.32, *P* < 0.0001)] in the average nucleus to anterior (N-A) distance was observed following induction (Fig. 5F and G and data not shown; Table 1). The very significant increase in mean N-A distance, and decrease in mean N-P distance despite the active microtubule growth at the posterior end (which would normally increase N-P) clearly demonstrate a dramatic re-positioning of the nucleus towards the posterior pole of the parasite.

The appearance of the nucleus positioning defect was examined in more detail to determine whether the defect observed first occurred in cells of a particular cell cycle stage (indicating the observed defect was due to a specific cell cycle defect), or whether it appeared in cells of all cell cycle stages simultaneously (indicating a general nucleus positioning defect unrelated to cell cycle stage). A lineage analysis over the first 24 h of *TbAIR9* RNAi induction was performed, analysing > 200 of each cell type (i.e. 1N1K, 1N2K and 2N2K cells) at each time point. Cells with abnormally positioned nuclei were observed for all cell cycle stages at all time points tested (including time 0, indicating some leaky expression of the RNAi) and increased in abundance for all cell cycle stages over time, suggesting the positioning defect occurred independently of cell cycle stage (Fig. S5).

### TbAIR9 is expressed in tsetse life cycle stages

The depletion of TbAIR9 from procyclic trypanosomes resulted in the nucleus being positioned posterior to the kinetoplast, giving a relative N/K positioning reminiscent of epimastigote life cycle stages in the tsetse fly (Sharma *et al*., 2008). To determine whether depletion of TbAIR9 is required for nucleus repositioning during differentiation from procyclic trypomastigote to epimastigote, expression of tyGFP:TbAIR9 from the *TbAIR9* endogenous locus was analysed in the tsetse transmissible strain of *T. brucei* 427, as epimastigotes cannot be cultured *in vitro.* Flies were dissected over a time-course to recover different trypanosome developmental stages, including asymmetric dividing cells [the transition stage from trypomastigote to epimastigote (Van den Abbeele *et al*., 1999; Sharma *et al*., 2008)]. All developmental stages analysed expressed tyGFP:AIR9, which appeared to be localized to the sub-pellicular cytoskeleton, but not to the flagellum (Fig. S6), as previously observed in culture forms (Figs 1 and S1). There was no noticeable reduction in tyGFP:AIR9 expression in any life cycle stage in the tsetse fly and hence, downregulation of TbAIR9 does not appear to be required for nucleus/kinetoplast repositioning during differentiation to epimastigotes.

### TbAIR9 is required for accurate cytokinesis in bloodstream trypanosomes, but depletion of TbAIR9 does not affect nucleus positioning

**Fig 6:**
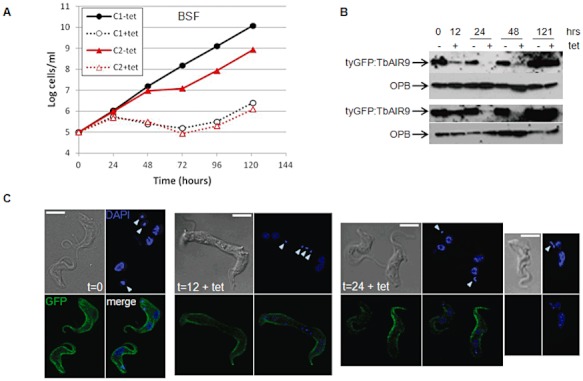
Depletion of *TbAIR9* in bloodstream *T. brucei* arrests growth. A. Cumulative growth curve of two independent *TbAIR9* RNAi cell lines expressing tyGFP:TbAIR9 from one endogenous allele in the presence (+ tet) or absence (− tet) of tetracycline. B. Western blot of lysates of *TbAIR9* RNAi cell lines expressing tyGFP:TbAIR9 from one endogenous allele, probed with anti-GFP antibody or with anti-OPB antiserum as a loading control. Upper blots: clone 1; lower blots: clone 2. Samples of cells grown in the presence or absence of tetracycline were taken at the time points indicated. C. Fluorescence microscopy images of *TbAIR9* RNAi cell lines expressing tyGFP:TbAIR9 from one endogenous allele. Cells were prepared for imaging at the time points (in hours) indicated. At 24 h post induction, the majority of cells had only a very faint tyGFP:TbAIR9 signal (see left cell in first panel set) – a few had significant staining (right cell in first panel set) and a few had no signal at all (second panel set). Top left: DIC images; top right: DAPI staining (blue); bottom left: tyGFP:TbAIR9 (green); bottom right: GFP/DAPI merge. Arrowheads point to kinetoplasts. Scale bars: 5 µm.

**Fig 7:**
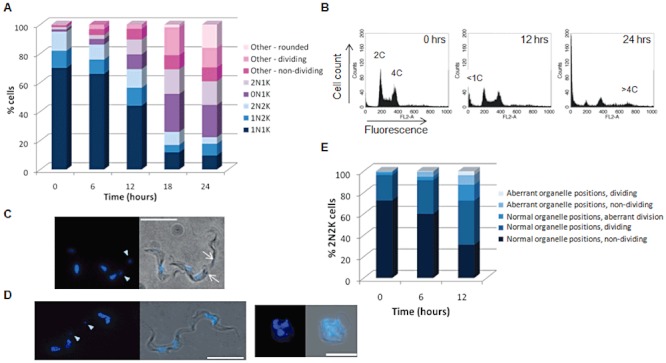
Depletion of TbAIR9 from bloodstream parasites impairs cytokinesis. A. DAPI staining of *TbAIR9* RNAi cells at the time points indicated, following induction. The number of nuclei (N) and kinetoplasts (K) in > 200 cells/time point are indicated. ‘Other’ comprise mainly multinucleate cells. ‘Dividing’ cells were those with a visible cleavage furrow, or those in abscission; ‘non-dividing’ cells had no visible furrow and were not in abscission. B. DNA content (flow cytometry) analysis of *TbAIR9* RNAi cells at the time points indicated, following induction. The ploidies of the peaks are indicated. C. Image of DAPI-stained 2N2K cell, undergoing cytokinesis to result in 0N1K and 2N1K progeny (left: DAPI; right: DAPI/DIC merge). Arrowheads point to kinetoplasts. D. DAPI stained images [as for (C)] of a dividing multinucleate cell (left) and a rounded multinucleate cell (right). Scale bars: 10 µm. E. Prevalence of nuclei and kinetoplast positioning and cytokinesis defects in 2N2K cells. Data presented are for clone 2.

To determine whether TbAIR9 performs similar functions in bloodstream stage trypanosomes, RNAi cell lines were generated in this life cycle stage, and characterized as described above. Induction of the RNAi response resulted in a growth defect visible from 24 h post induction (Fig. 6A). Cells began to die from 24 h post induction, but normal growth rates started to resume from 72–96 h post induction. Depletion of TbAIR9 was confirmed by replacing one *TbAIR9* allele with *tyGFP:TbAIR9* in two independent RNAi clones and analysing tyGFP:TbAIR9 protein levels by Western blotting (Fig. 6B). TbAIR9 levels were reduced by 12 h post induction and were substantially depleted by 24 h post induction, but recovered by 121 h post induction, indicating that the resumed normal growth rates in these cell lines were due to the appearance of RNAi revertants re-expressing AIR9, as has been previously described (Chen *et al*., 2003). Depletion of TbAIR9 following RNAi induction was also confirmed by fluorescence microscopy (Fig. 6C). Similar to procyclic RNAi cell lines, tyGFP:TbAIR9 appeared to be preferentially depleted from the posterior end of the parasite and by 24 h post induction, most cells only showed a faint tyGFP:TbAIR9 signal and a few cells (∼5%) had lost their tyGFP:TbAIR9 signal completely. Cell cycle analysis (note that both clones showed similar results, so only data for one clone is presented here) revealed that depletion of TbAIR9 resulted in the accumulation of zoids (0N1K) and 2N1K cells from 6 h post-RNAi induction (Fig. 7A and B). These cell types arose in approximately equal numbers, suggesting that they were the products of an aberrant division of a common 2N2K progenitor cell. Indeed, 2N2K cells in the process of dividing to give 2N1K and 0N1K daughter cells were observed (Fig. 7C). Over time, zoids continued to increase in abundance, reaching 20–40% of the total population by 24 h post induction (Fig. 7A). 2N1K cells increased to about 10% of the total population at 18 h, but did not increase further in abundance, probably because as discussed above, they were converted to multinucleate cells. Abnormal cells, containing multiple nuclei and kinetoplasts, were visible in small numbers from 6 h post induction but reached 25–40% of the total cell population by 24 h post induction (Fig. 7A), and a > 4C peak appeared in flow cytometry profiles at this time point (Fig. 7B). Many of these cells were in the process of furrowing or undergoing abscission, and some had a rounded morphology (Fig. 7A and D).

Cells with normal N/K configurations were examined to determine whether, like the procyclic form, depletion of TbAIR9 resulted in cell lengthening or abnormal positioning of the nucleus and kinetoplast. Cell length was not significantly different in induced cells compared with uninduced cells (data not shown), suggesting depletion of AIR9 did not result in increased microtubule extension as it did in procyclic cells. Abnormal relative positioning of the nucleus and kinetoplast was only observed in some 2N2K cells, and not in 1N1K or 1N2K cells (data not shown). 2N2K cells (mostly those with normal N/K positions but additionally some with abnormally positioned nuclei and kinetoplasts) were also observed to undergo defective cytokinesis, yielding non-equivalent progeny (Fig. 7C and E).

## Discussion

This study describes the characterization of TbAIR9, showing it to be a novel CAP in *T. brucei*. Two different epitope-tagged TbAIR9 proteins, expressed from the endogenous *TbAIR9* locus, colocalized and fractionated with tubulin in the subpellicular microtubules but not in the flagellum. The lack of observed TbAIR9 localization to the flagellum contrasts with previous proteomic studies, which have identified TbAIR9 in flagellar fractions (Broadhead *et al*., 2006; Hart *et al*., 2009; Zhou *et al*., 2010), and may indicate that TbAIR9 was a contaminant in these proteomes. Consistent with this, CAP5.5, which by immunofluorescence, like TbAIR9, localizes to the cell body but not the flagellum (Hertz-Fowler *et al*., 2001), was also identified as a component of the flagellar proteomes in these studies.

Our data do not allow us to discriminate whether TbAIR9 directly interacts with subpellicular microtubules or whether TbAIR9 associates with the cytoskeleton through interaction with a MAP. TbAIR9 lacks the AtAIR9 microtubule binding domain, but another region of the protein could potentially perform this function in *T. brucei*, and experiments are in progress to map the domain(s) responsible for cytoskeleton association. Following RNAi induction, tyGFP:AIR9 behaves like the MAPs, WCB and CAP5.5 (Baines and Gull, 2008; Olego-Fernandez *et al*., 2009), being depleted preferentially from the posterior end of the cell where microtubules are most dynamic. The increase in microtubule extension at the posterior end of the cell following TbAIR9 depletion may suggest that TbAIR9 influences microtubule dynamics, at least in procyclic trypanosomes, but since depletion of TbAIR9 did not appear to grossly alter the structure of the cytoskeleton, it is unlikely to be a key structural component or to function in the cross-linking of microtubules to each other or to the plasma membrane.

Functional characterization of TbAIR9 indicated that it is essential for normal proliferation in both bloodstream and procyclic life cycle stages. In procyclic trypanosomes, depletion of TbAIR9 profoundly affected the positioning of the nucleus within the cell, resulting in its relocation to the posterior pole, while only minimal movement of the kinetoplast occurred. Aberrant nucleus positioning was observed from 24 h post induction, prior to the substantial accumulation of cells with 0N1K and 2N1K and other aberrant N/K configurations. Hence, while it is possible that TbAIR9 is important for cytokinesis in this life cycle stage, these cell division defects could have occurred as a downstream consequence of defective nucleus positioning, rather than due to an a *priori* defect in cytokinesis. Migration of the nucleus and kinetoplast occurs naturally during differentiation events in the trypanosome life cycle. During differentiation from the stumpy bloodstream form to the procyclic form, active microtubule extension at the posterior end of the cell results in the kinetoplast migrating anteriorly from the posterior end of the cell to approximately midway between the nucleus and the posterior end (Matthews *et al*., 1995). Subsequently, during differentiation of the procyclic trypomastigote to the epimastigote life cycle stage via the asymmetric dividing form, the nucleus moves substantially towards the posterior of the cell, so that it is located posterior to the kinetoplast (Sharma *et al*., 2008). Thus, in terms of relative nucleus and kinetoplast positions, TbAIR9-depleted procyclic trypanosomes resemble epimastigote forms. However, since TbAIR9 is expressed throughout the developmental cycle in the tsetse fly, it does not appear that depletion of TbAIR9 is required for differentiation to an epimastigote, although this does not rule out that TbAIR9 is perhaps instead functionally altered by a post-translational modification during this differentiation process.

To date, nothing is known about how the position of the *T. brucei* nucleus is determined or maintained in the cell throughout the cell cycle. In *Chlamydomonas reinhardtii*, fibres containing centrin link the basal bodies to the nucleus and contract during mitosis, displacing the nucleus towards the flagellum/basal bodies (Wright *et al*., 1985; Salisbury *et al*., 1988). Nucleus positioning may also be controlled by either actin or microtubule dynamics and forces. In *A. thaliana*, repositioning of nuclei in response to light requires actin bundles associated with the nucleus (Iwabuchi and Takagi, 2010), and Nesprin 1 and Anc-1, which provide a link between the nuclear envelope and actin filaments, are required for nucleus tethering in mammals and *Caenorrhabditis elegans* respectively (Starr and Han, 2002; Zhang *et al*., 2010). Alternatively, nuclei can be displaced along microtubules by cytoplasmic dynein and kinesins (Reinsch and Karsenti, 1997; Gonczy *et al*., 1999), with kinesin-1 being responsible for forward movement of nuclei and dynein moving nuclei backwards short distances to bypass obstacles in the cell (Fridolfsson and Starr, 2010), or can be pushed or pulled via astral microtubules (Burke and Roux, 2009). However, in *T. brucei*, depletion of centrins (He *et al*., 2005; Selvapandiyan *et al*., 2007; Shi *et al*., 2008), actin (Garcia-Salcedo *et al*., 2004) or downregulation of kinesins KIN13-1, KIN13-2, KIF9A or KIF9B (Demonchy *et al*., 2009; Chan and Ersfeld, 2010; Chan *et al*., 2010; Wickstead *et al*., 2010) does not appear to affect nucleus positioning, while the function of cytoplasmic dynein has not been investigated. No physical linkages between the nucleus and the microtubule cytoskeleton have been visualized in electron microscopy studies to date, but since parts of the nuclear envelope are located in close proximity to the cytoskeleton, it is possible that CAPs could bridge to the nucleus. Our data suggest that TbAIR9 and/or its, as yet unidentified, binding partners could be involved in tethering the nucleus to the cytoskeleton.

In contrast to the procyclic form, depletion of TbAIR9 did not cause dramatic changes to nucleus positioning in the bloodstream stage, which could reflect differences in nucleus dynamics in this life cycle stage, since nucleus segregation following mitosis is less extensive in bloodstream stage trypanosomes (Tyler *et al*., 2001). Depletion of TbAIR9 in the bloodstream stage did, however, result in defects in cytokinesis, as 2N2K cells divided unequally to give 2N1K and 0N1K cells and multinucleate/multikinetoplast cells appeared in the population at later time points. The generation of 2N1K and 0N1K daughter cells is likely to have arisen from an aberrantly positioned cleavage furrow, since at abscission, the daughter cell bodies were often of very unequal size, with the zoid being considerably thinner than the 2N1K cell (see Fig. 7C), indicating that the furrow had not progressed along the midline of the cell. Since TbAIR9 outlines the daughter cell body prior to cytokinesis, it may therefore contribute to the positioning of the furrow, ensuring that it tracks correctly.

In summary, our data reveal that certain features of AIR9 are conserved in *T. brucei* and *A. thaliana*. AIR9 is associated with the microtubule cytoskeleton in both organisms, and is important for cytokinesis. However, TbAIR9 appears to have an additional novel role in controlling nucleus positioning in procyclic trypanosomes. We hypothesize that TbAIR9 may therefore, perhaps via its A9 immunoglobulin-like domains, interact with proteins that control cytoskeletal dynamics, nucleus positioning and cell division in the parasite. Future identification of TbAIR9 binding partners should shed light on the regulation of these processes. However, the differences in phenotypes observed following RNAi-mediated knockdown of TbAIR9 in bloodstream and procyclic trypanosomes emphasize the fundamental differences in cell cycle regulation and the cytoskeletons of these two life cycle stages.

## Experimental procedures

### Culturing and transfection of trypanosomes

*Trypanosoma brucei brucei* strain Lister 427 (procyclic and bloodstream stages) and the RNAi cell lines 427 pLew13 pLew29 (procyclic form, ‘29-13’) and 427 pLew13 pLew90 (bloodstream stage, ‘90-13’) (Wirtz *et al*., 1999) were cultured and transfected as described previously (Hammarton *et al*., 2003; Burkard *et al*., 2007). For tsetse fly studies, the fly transmissible *T. brucei brucei* 427 variant 3 (MOVS/UG/60/427) was cultured and transfected as described in (Peacock *et al*., 2008).

### Analysis of tsetse life cycle stages

Tsetse flies were maintained at 25°C and 70% relative humidity and fed via a silicone membrane. The first feed 24–48 h post eclosion consisted of approximately 10^7^ procyclic trypanosomes per ml of washed horse red blood cells, supplemented with 10 mM l-glutathione (Macleod *et al*., 2007) to increase infection rates. Thereafter flies were maintained on sterile defibrinated horse blood supplemented with 1 mM dATP. Flies were dissected 3–32 days post infection, removing separately the proventriculus, the alimentary tract from the anterior midgut to the rectum, and the salivary glands. Each body part was placed in 10 µl of phosphate-buffered saline (PBS), before being teased apart and allowing trypanosomes to settle onto glass slides. Samples were fixed in 1% PFA for 20 min in a humid chamber, washed three times in PBS and allowed to dry briefly before being stained and mounted with the DNA stain 4′,6-diamidino-2-phenylindole (DAPI) in Vectashield and visualized by fluorescence microscopy using a DMRB microscope (Leica) equipped with a Retiga Exi camera and Velocity version 4.1 software (Improvision). Trypanosome life cycle stages were identified according to Sharma *et al*. (2008).

### Epitope tagging of AIR9 at the endogenous locus

Cell lines expressing TbAIR9 tagged with tyGFP at the N-terminus (tyGFP:TbAIR9) from the *TbAIR9* endogenous locus were generated by transfecting Lister 427 cells with plasmid pHG172. pHG172 was generated by polymerase chain reaction (PCR)-amplifying the 269 bp upstream of the *TbAIR9* start codon (5′ UTR fragment) with PR94 and PR95 (incorporating XbaI and NheI restriction sites respectively; Table S1) and bases 4–358 of the *TbAIR9* open reading frame (ORF; accession number FR750404) with PR96 and PR97 (incorporating NheI and BclI restriction sites respectively; Table S1) from Lister 427 genomic DNA, cloning them into pSC-B (Stratagene) to facilitate sequencing (performed by The Sequencing Service, University of Dundee) and then subcloning them using the above restriction sites in a three-way ligation into pENT-6-Blast eGFP-TY (Kelly *et al*., 2007) digested with XbaI and BamHI. pHG172 was linearized by NheI digestion prior to transfection. Lister 427 cell lines were also transfected with pHG182 to generate cells that expressed TbAIR9 tagged with 6xHA epitopes at the C-terminus (TbAIR9:6ha) from the endogenous locus. pHG182 was generated by PCR-amplifying bases 2253–2979 of *AIR9* with PR228 and PR229 (incorporating HindIII and XbaI restriction sites respectively; Table S1), which were cloned into pSC-B and sequenced, before being subcloned into HindIII/XbaI-cut pOrc-_6XHA_Ble (Tiengwe, 2010); a modification of pNAT^6myc^_x_ (Alsford and Horn, 2008), where the 6xMYC tag and the blasticidin resistance marker have been replaced with 6xHA and bleomycin resistance respectively). Expression of the tagged proteins *in vivo* was confirmed by 12% SDS-PAGE followed by Western blotting appropriate cell lysates (typically 10^6^ cells per lane) with a 1/50 dilution mouse anti-TY (Bastin *et al*., 1996), 1/2000 dilution rabbit anti-GFP (Santa Cruz) or 1/1000 dilution rat anti-HA (Roche) monoclonal antibodies. Plasmid pHG172 was also transfected into the 427 variant 3 procyclic cell line, and after confirmation of tyGFP:TbAIR9 fluorescence, the resultant cell line was used for tsetse transmission studies.

### Generation and induction of RNAi cell lines

A 413 bp fragment of the *TbAIR9* ORF [bases 1803–2215, identified by the RNAit software (Redmond *et al*., 2003)], to be unique, was amplified from Lister 427 genomic DNA using OL2696 and OL2697 (incorporating HindIII and BamHI restriction sites respectively; Table S1) and cloned into the same sites of p2T7_ti_:GFP vector (LaCount *et al*., 2000) in place of the *GFP* coding sequence, generating pHG27. Plasmid pHG27 was linearized with NotI and transfected into the RNAi cell lines as above. The RNAi response was induced by the addition of 1 µg ml^−1^ tetracycline to the culture medium. Two independent clonal RNAi cell lines for each life cycle stage selected for downstream analyses were also transfected with pHG172 to allow monitoring of AIR9 downregulation following RNAi induction. Population doubling times were calculated for exponential phase growth, using the equation *g* = 0.301*t*/log*N_t_* − log*N*_0_, where *g* is the population doubling time, *t* is the time in hours and *N_t_* and *N*_0_ represent cell numbers at time *t* and time 0 respectively.

### Subcellular fractionation

To prepare subcellular fractions, *T. brucei* cells were washed once in PBS (procyclic form) or trypanosome dilution buffer [TDB (Forsythe *et al*., 2009)] (bloodstream form) before being resuspended in ice cold PEME buffer (100 mM PIPES, 2 mM EGTA, 1 mM MgSO_4_, pH 6.9) supplemented with 1% Nonidet P40 and 1× complete EDTA-free protease inhibitor cocktail (Roche) and incubated on ice for 2 min before being centrifuged at 800 *g* for 10 min to separate cytosolic proteins (supernatant, S1) from the cytoskeleton (pellet, P1). Cytoskeletons were resuspended in ice cold high salt buffer (PEME supplemented with 1 M NaCl, protease inhibitors, 200 µg ml^−1^ DNase I and 50 µg ml^−1^ RNase A) and incubated on ice for 10 min before being centrifuged at 16 000 *g* for 15 min at 4°C to separate extracted MAPs and tubulin (supernatant, S2) from the remaining cytoskeleton (pellet, P2). The cytoskeletal pellet was treated once more with high salt buffer and centrifuged for a second time to extract any remaining non-flagellar MAPs and tubulin (supernatant, S3), leaving flagella in the pellet (P3). Samples were analysed by SDS-PAGE and Western blotting with appropriate antibodies. To check the quality of the subcellular fractionation, fractions were probed with antibodies against the cytosolic proteins EF1α (1/25 000 dilution; Millipore) or oligopeptidase B [OPB, 1/1000 dilution (Munday *et al*., 2011)], the cytoskeletal protein β-tubulin (KMX antibody, 1/4000 dilution; Millipore) and the flagellar proteins PFR1/2 (L13D6 antibody, 1/1000 dilution; Kohl *et al*., 1999). To analyse trypanosome cytoskeletons by microscopy, washed cells were settled onto poly-lysine coated glass slides, and cytoskeletons generated by incubating in PEME/1% NP40 for 1 min (procyclic form) or PEME/0.75% NP40 for 30 s (bloodstream form), prior to washing in PBS and fixing in −20°C methanol for 30 min. Cytoskeletons were stained with DAPI and analysed by fluorescence microscopy (see below).

### Fluorescence microscopy and flow cytometry

DAPI staining of fixed cells, flow cytometry of propidium iodide-stained cells and immunofluorescence with the YL1/2 antibody (SantaCruz) were performed as previously described (Hammarton *et al*., 2003; 2004). Fluorescence microscopy was performed using either an Axioskop fluorescent microscope (Zeiss) and OpenLab version 5.5 software or with a DeltaVision RT Epifluorescence Imaging system (Applied Precision) and SoftWoRx software, with cells viewed under appropriate filter sets. For immunofluorescence of tagged AIR9 cell lines, cells were fixed in 1% formaldehyde for 30 min at room temperature, washed twice in PBS and permeabilized by incubation in 1% Triton X-100 in PBS for 5 min. Cells were then incubated in 1 M glycine in PBS for 10 min followed by a 1/100 dilution of rat anti-HA (Roche) in 0.1% Triton X-100, 0.1% BSA for 1 h. Cells were then washed in PBS before being incubated in a 1/100-1/1000 dilution of an appropriate AlexaFluor dye-conjugated secondary antibody (Invitrogen) for 1 h. Cells were washed again, stained with DAPI and viewed under UV light as described above.

### Measurement of cellular dimensions

Cells were co-stained with the anti-β-tubulin antibody, KMX (Millipore), and DAPI and the following dimensions measured using OpenLab version 5.5 software: total cell length (L), centre of kinetoplast to posterior end (K-P), posterior edge of nucleus to posterior end (*N_i_*) and anterior edge of nucleus to posterior end (*N_ii_*). The centre of the nucleus to the posterior end (N-P) dimension was calculated as (*N_i_* + *N_ii_*)/2, and K-A and N-A were calculated as total cell length minus K-P and N-P respectively. *F*-tests were performed to determine whether the variances of each dimension set (for each clone, minus and plus tetracycline) were equal to allow an appropriate *t*-test to be performed. Following Bonferonni correction, *t*-tests were performed to compare uninduced and induced populations of each clone for each measured dimension.

### Transmission electron microscopy

Whole cells were fixed in 2.5% glutaraldehyde/0.1 M sodium cacodylate buffer for 1 h at room temperature before being washed three times in 0.1 M sodium cacodylate buffer and then post-fixed in 1% (w/v) osmium tetroxide/0.1 M sodium cacodylate buffer for 1 h. Cells were then washed three times in dH_2_O and dehydrated in an ethanol series before being incubated in propylene oxide/Epon resin overnight and then being embedded in pure Epon resin. Sections (60–70 nm) were contrast stained with aqueous 2% uranyl acetate and Reynold's lead citrate prior to being analysed by zero-loss imaging on a LEO 912 AB energy filtering TEM. Images were processed using iTEM imaging platform software (Olympus SIS).

Whole mount cytoskeleton preparations were prepared according to Sherwin and Gull (1989) with some modifications. Briefly, procyclic cells were washed in PBS and allowed to settle onto freshly glow discharged carbon-coated/Formvar-filmed 300 mesh copper grids for 5 min. Cells were then incubated in PEME buffer supplemented with 1% NP40 for 2 × 10 min to generate cytoskeletons. Cytoskeletons were post-fixed in 2.5% glutaraldehyde in PEME buffer for 10 min, washed twice in distilled water, negatively stained with 1% trehalose/2.5% ammonium molybdate and dried. Specimens were analysed on a FEI Tecnai 12 transmission electron microscope and images acquired using Gatan Digital Micrograph.
