# Supplementary material for: The Trypanosoma brucei AIR9-like protein is cytoskeleton-associated and is required for nucleus positioning and accurate cleavage furrow placement
Source: Mol Microbiol. 2012 Mar 5;84(1):77–92. doi: 10.1111/j.1365-2958.2012.08008.x (PMC3488599; doi:10.1111/j.1365-2958.2012.08008.x)
Supplement: Supplementary file 1 [file mmi0084-0077-SD1.pdf]

Figure S1

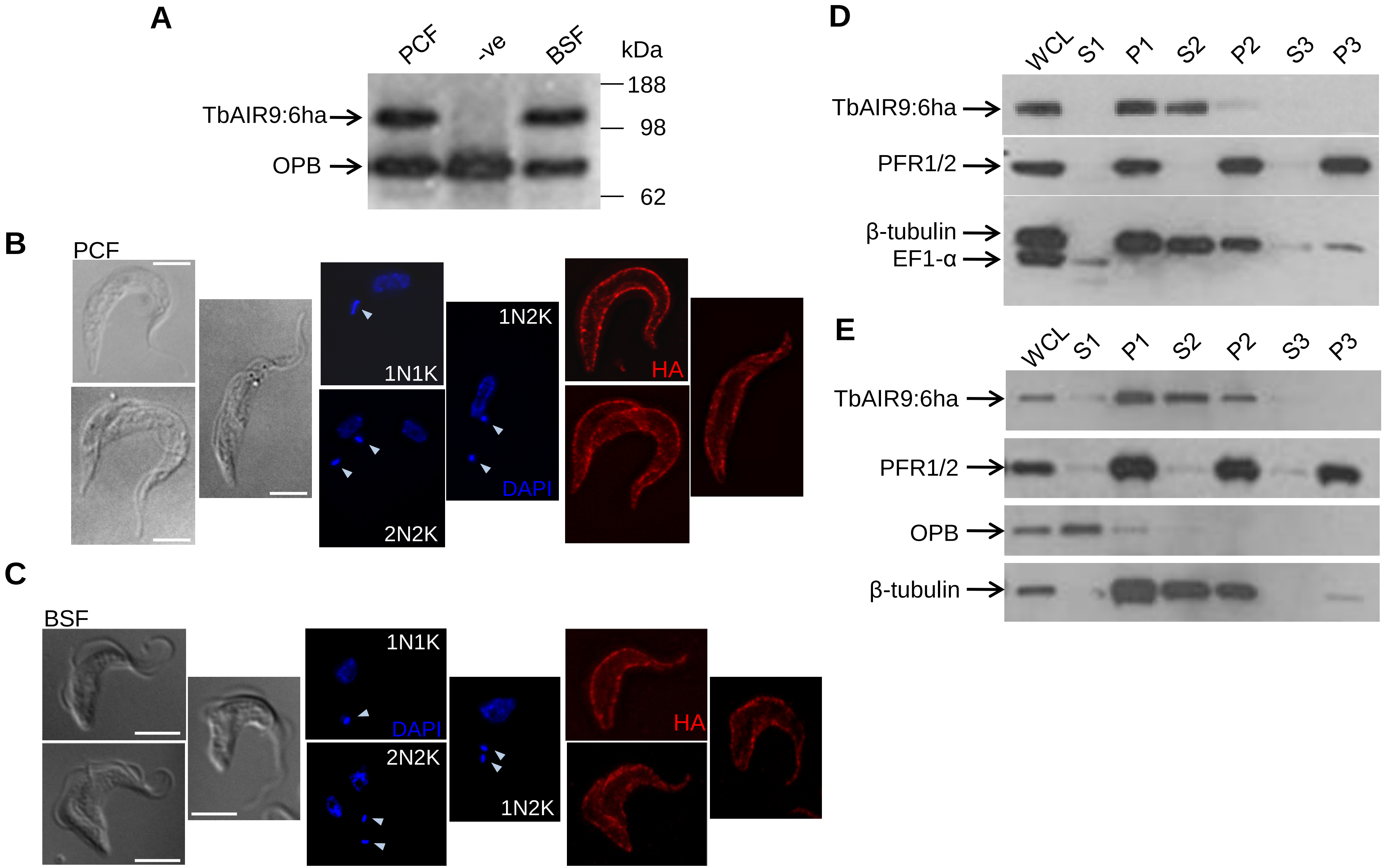
A
PCF
-ve
BSF
kDa
188
98
62
TbAIR9:6ha
OPB
B
PCF
1N1K
1N2K
2N2K
DAPI
HA
C
BSF
1N1K
DAPI
2N2K
1N2K
HA
D
WCL
S1
P1
S2
P2
S3
P3
TbAIR9:6ha
PFR1/2
β-tubulin
EF1-α
E
WCL
S1
P1
S2
P2
S3
P3
TbAIR9:6ha
PFR1/2
OPB
β-tubulin

Figure S2

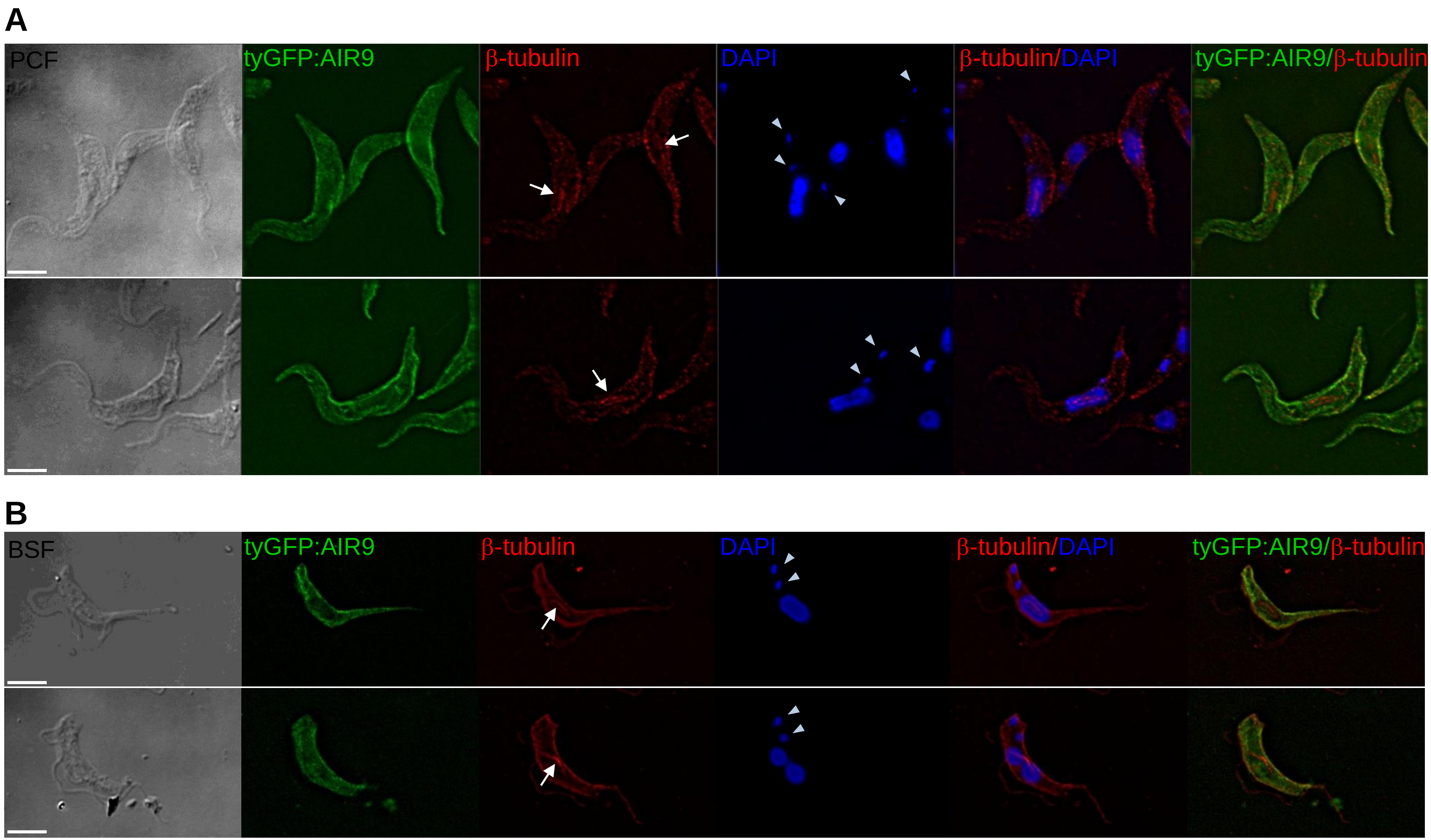
A
PCF
tyGFP:AIR9
β-tubulin
DAPI
β-tubulin/DAPI
tyGFP:AIR9/β-tubulin
B
BSF
tyGFP:AIR9
β-tubulin
DAPI
β-tubulin/DAPI
tyGFP:AIR9/β-tubulin

Figure S3

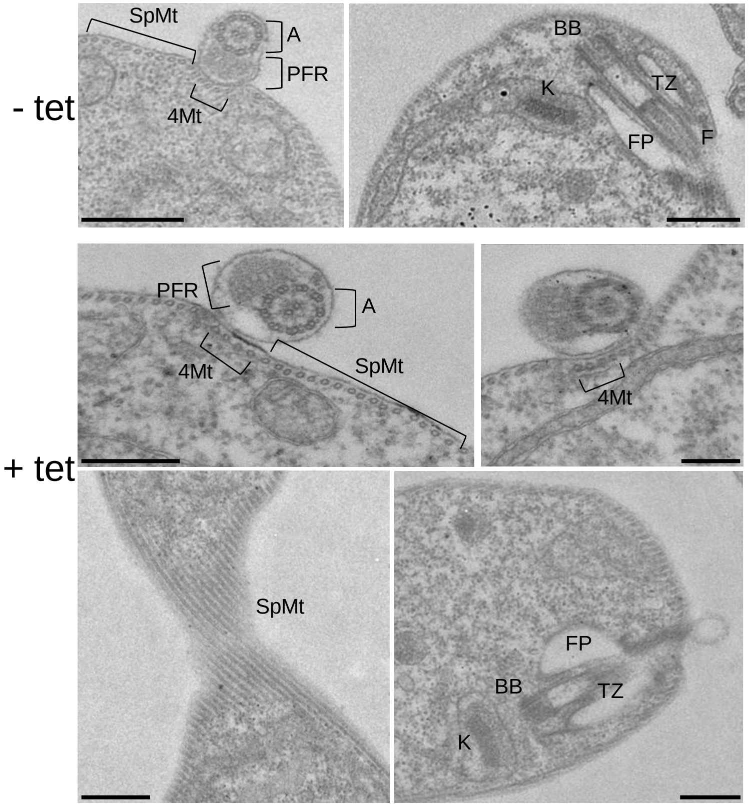

- tet
+ tet
SpMt
A
PFR
4Mt
BB
K
TZ
FP
F
PFR
A
4Mt
SpMt
4Mt
SpMt
FP
BB
TZ
K

Figure S4

A

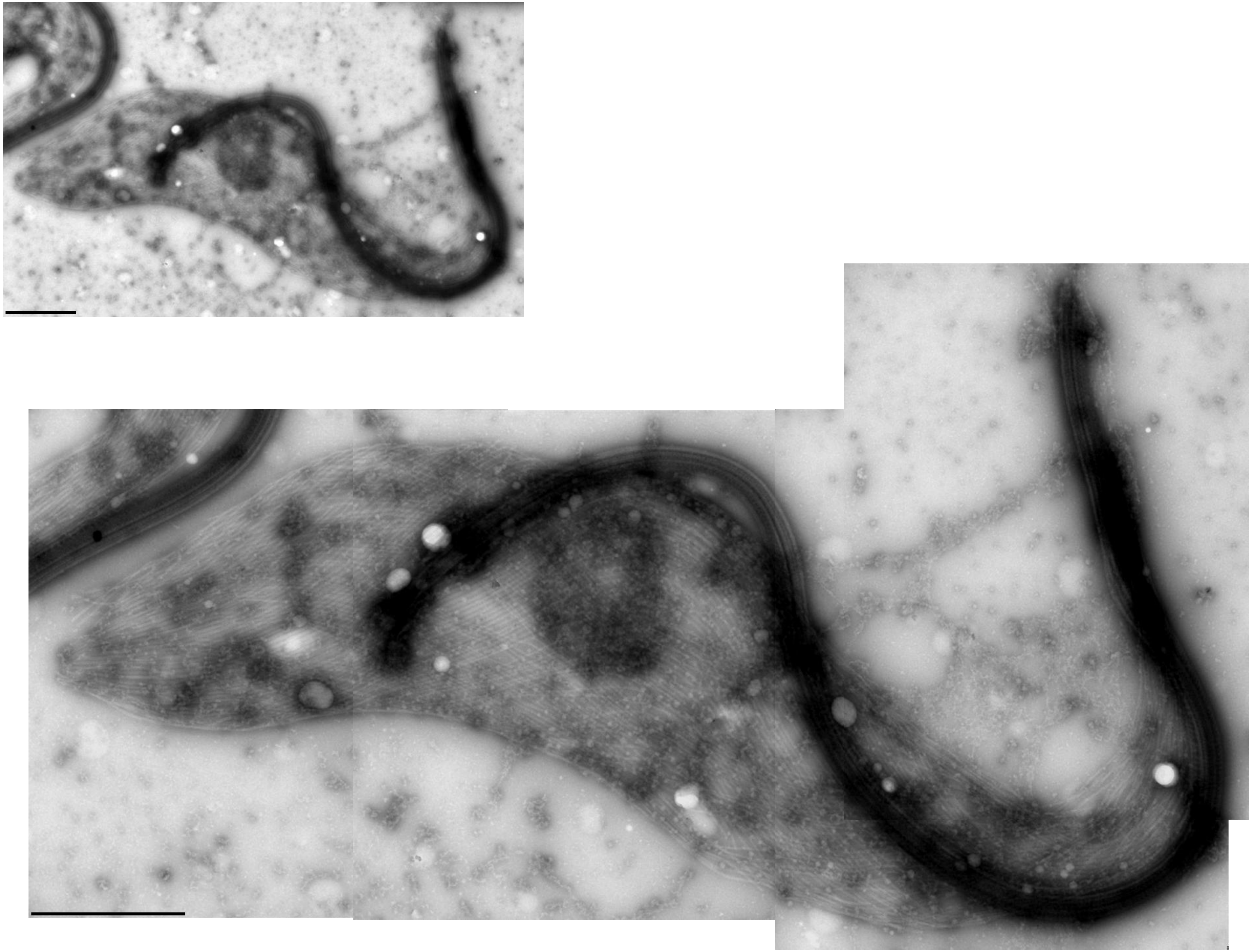

B

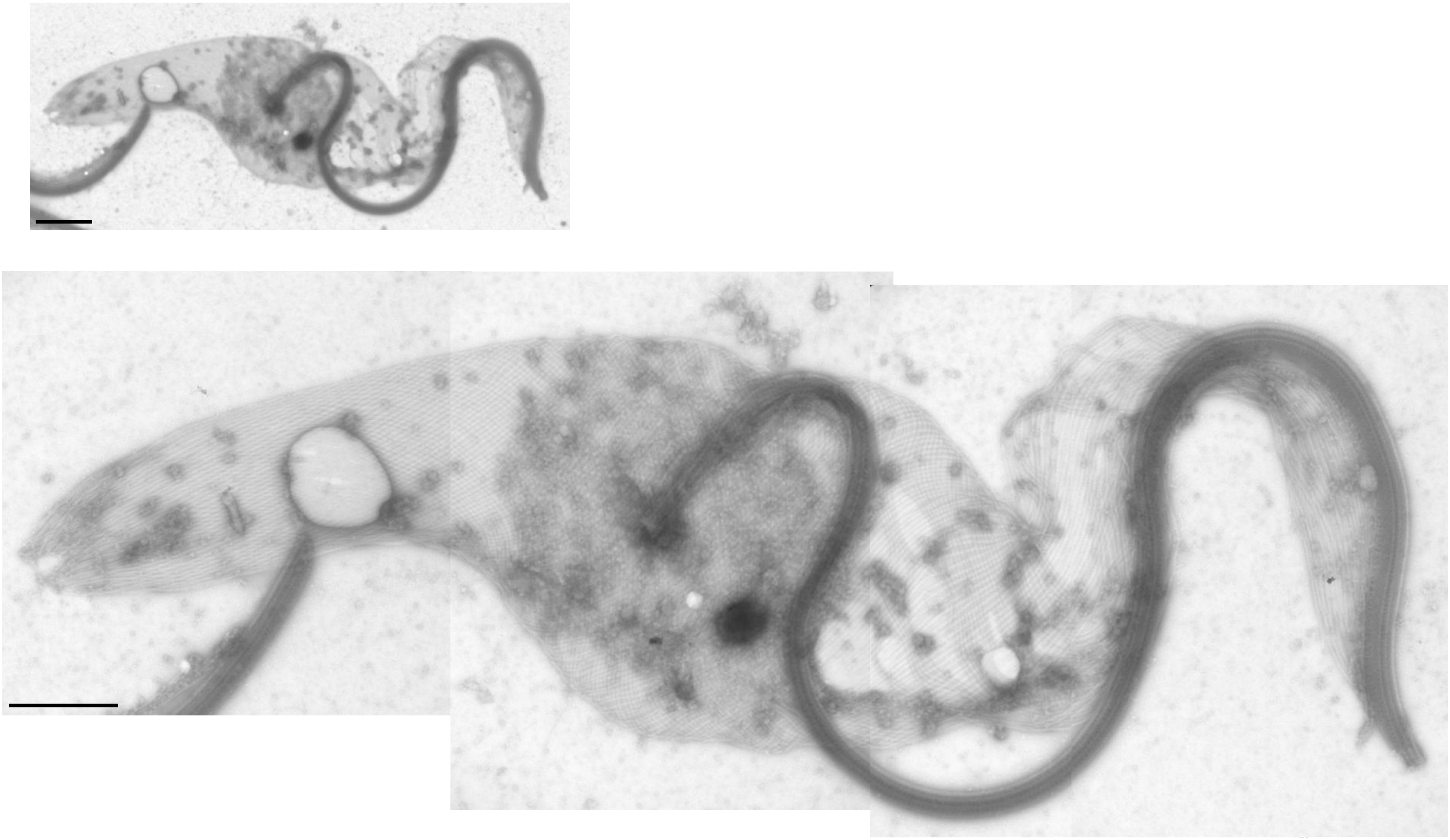

C

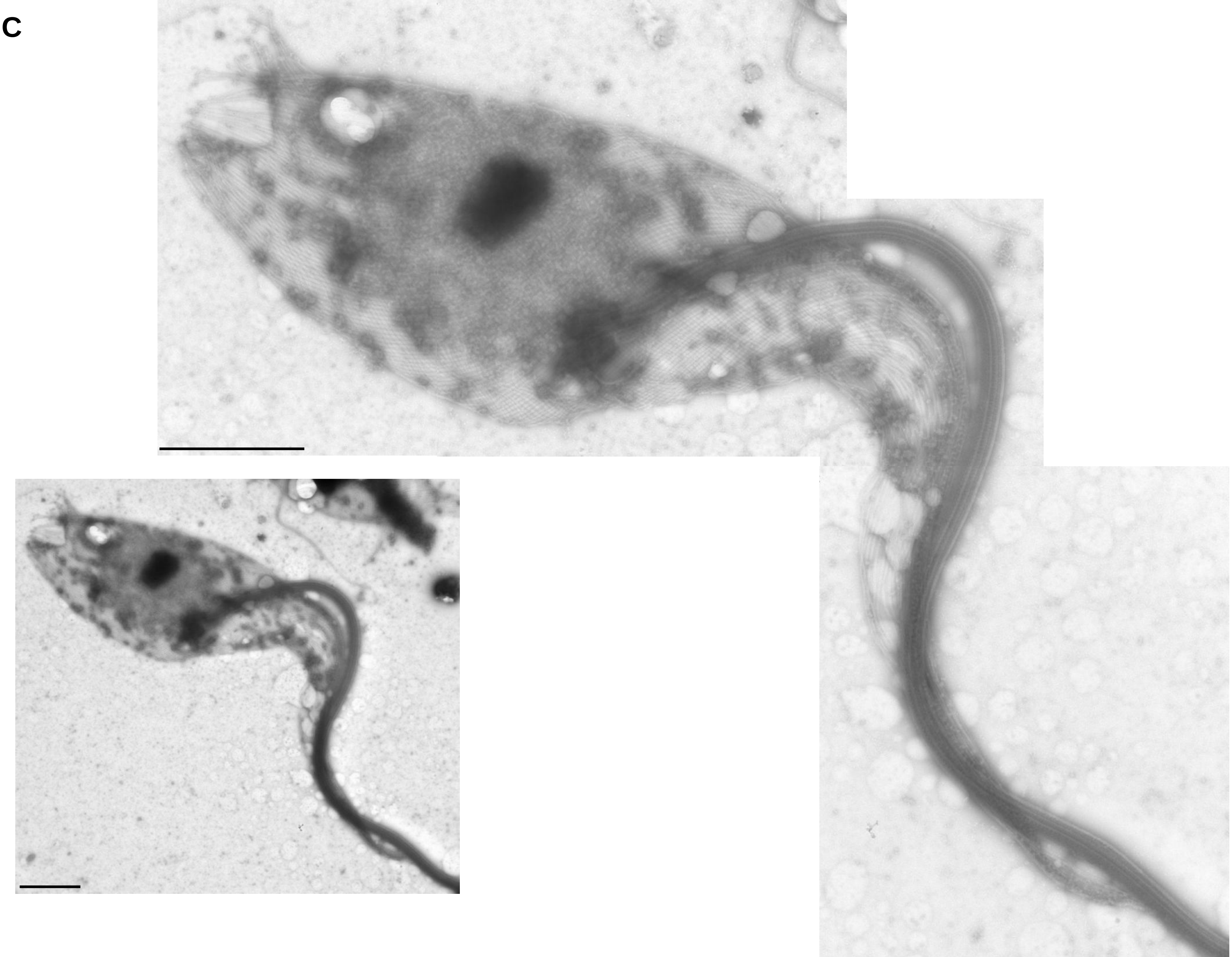

D

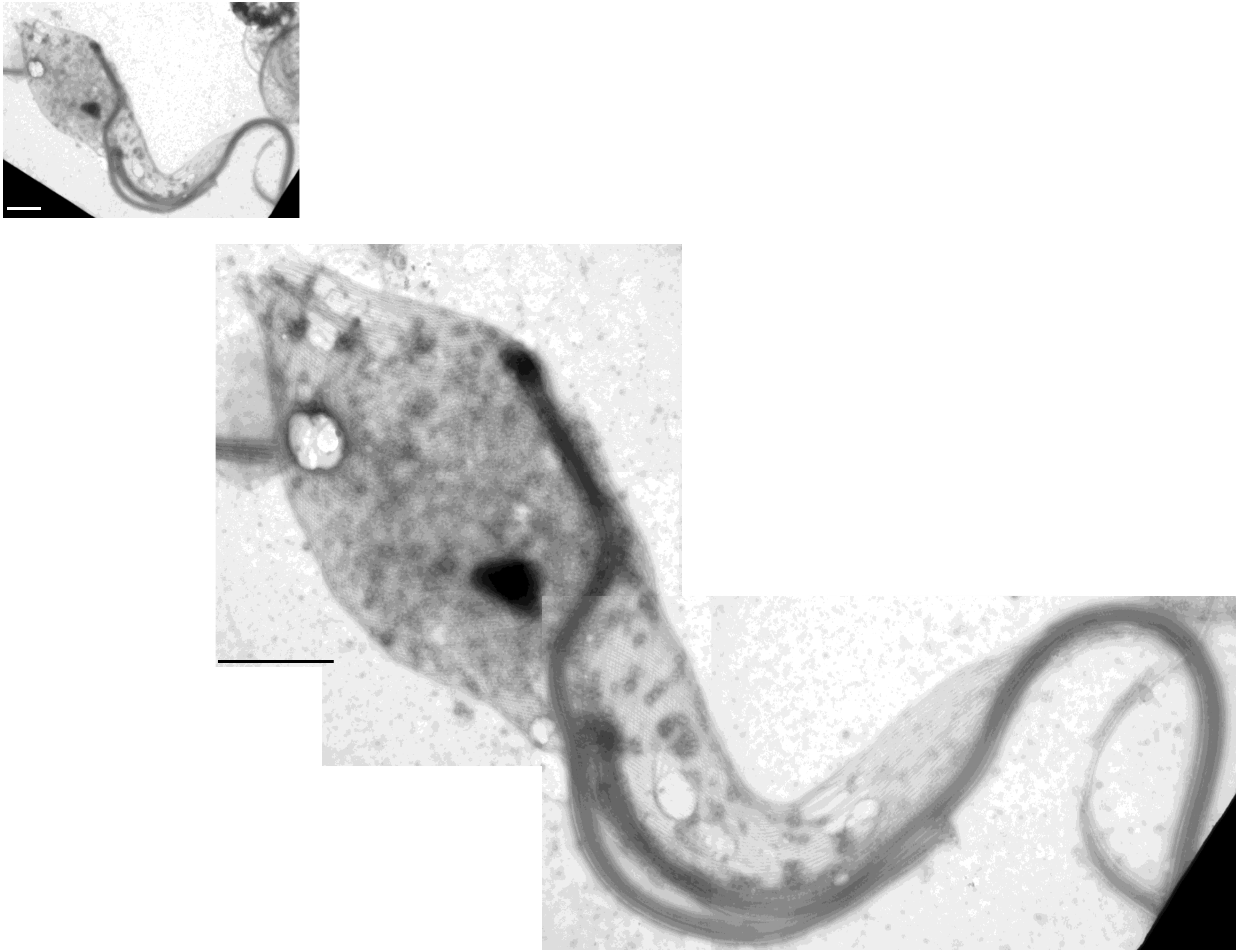

E

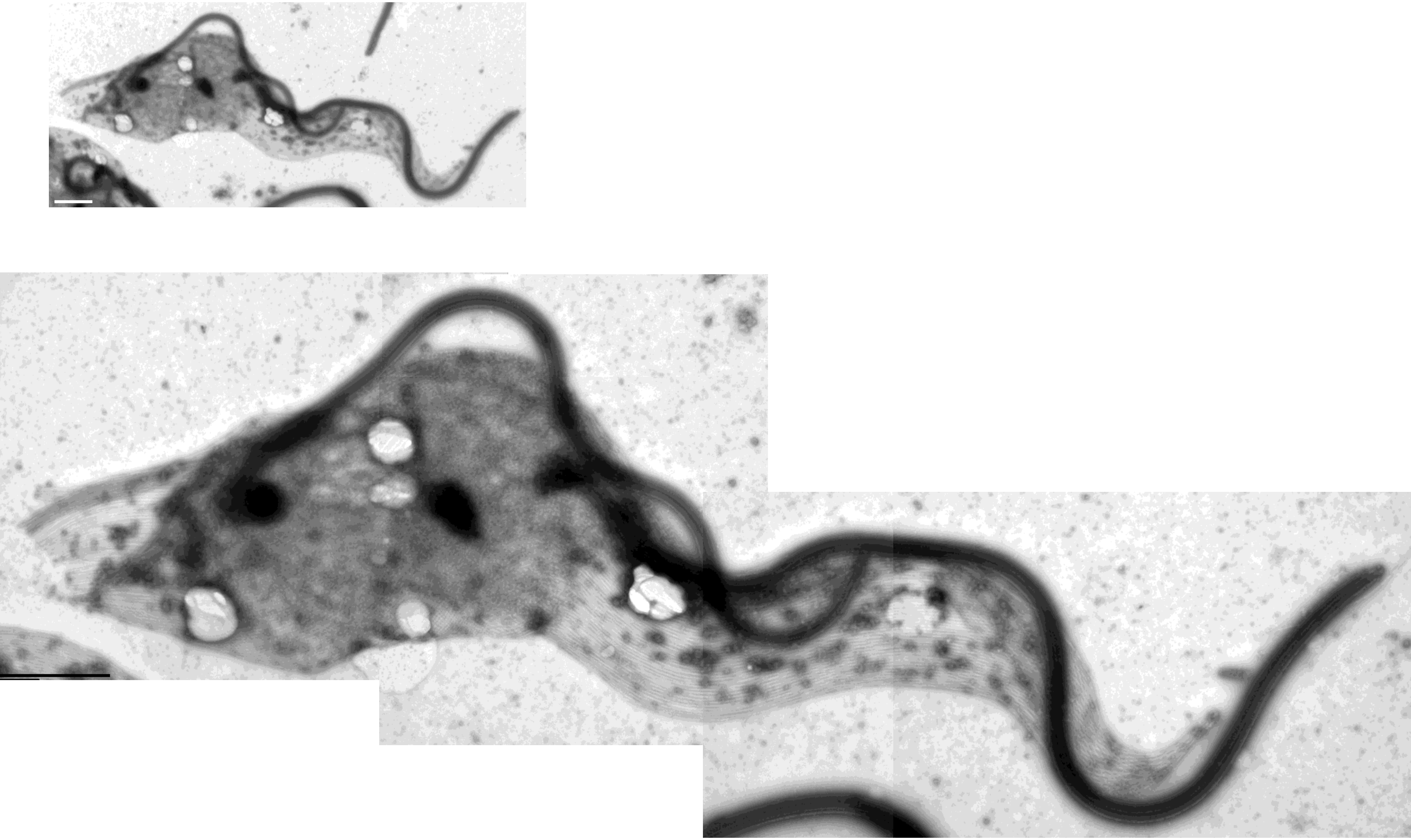

Figure S5

A

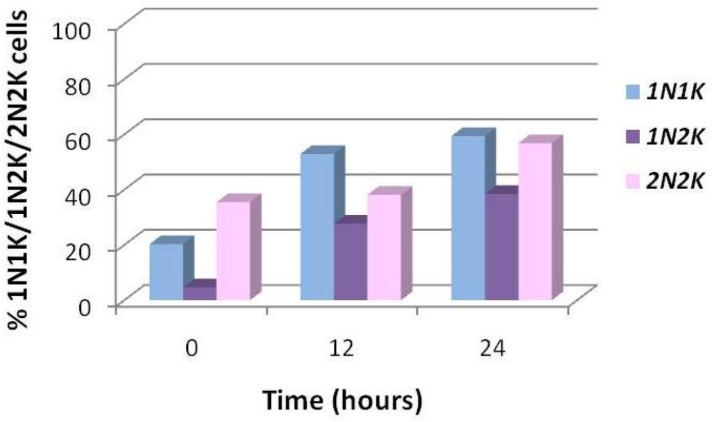

% 1N1K/1N2K/2N2K cells
100
80
60
40
20
0
0
12
24
Time (hours)
1N1K
1N2K
2N2K

B

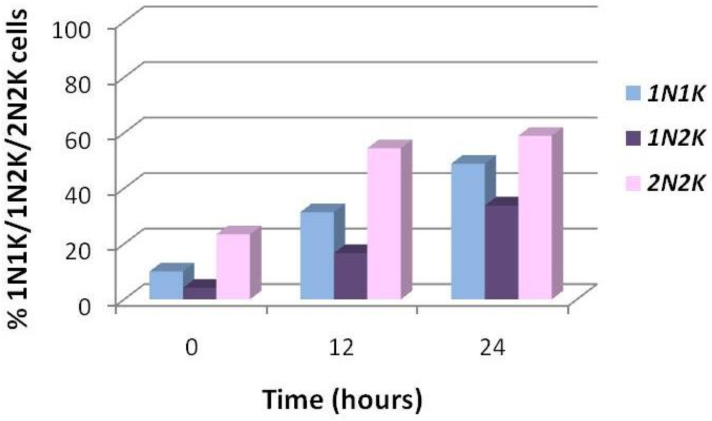

% 1N1K/1N2K/2N2K cells
100
80
60
40
20
0
0
12
24
Time (hours)
1N1K
1N2K
2N2K

Figure S6

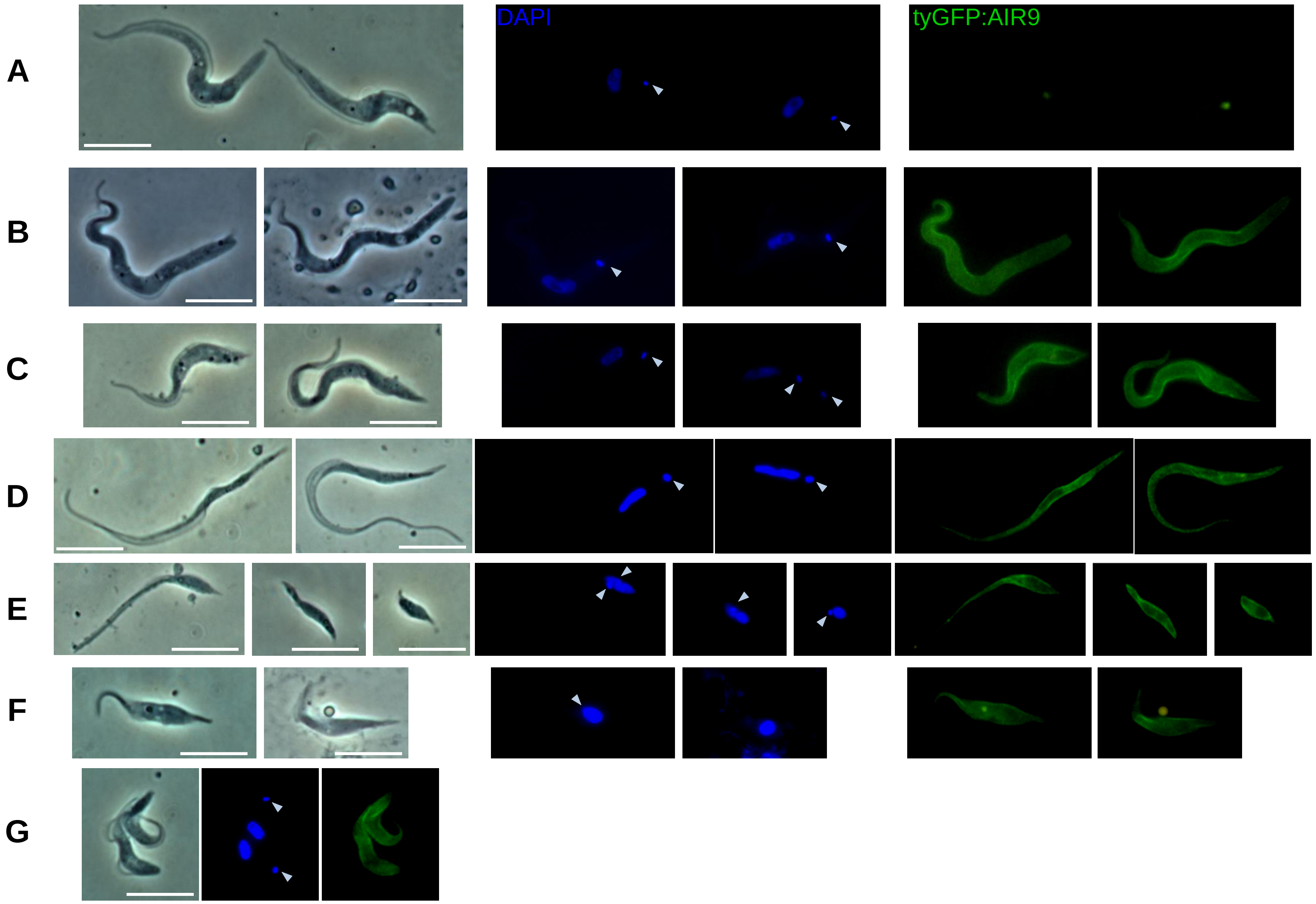

Table S1. Oligonucleotides used in this study

Details of the oligonucleotides used in this study are given below. The restriction site in the oligonucleotide is underlined.

**Oligo**	**Details**	**Restriction site**	**Sequence (5`-3`)**
PR94	Sense oligo for *TbAIR9* 5` UTR	*Nhe* I	CCTGCTAGCCGTCATCTTCG GTGGCAACA
PR95	Antisense oligo for *TbAIR9* 5` UTR	*Bcl* I	CGCTGATCACGTGTATACCA GAAATAACC
PR96	Sense oligo for *TbAIR9* fragment (bp 4-358)	*Xba* I	GCGTCTAGAAGTGCGTCTGC GATAAGGCG
PR97	Antisense oligo for *TbAIR9* fragment (bp 4-358)	*Nhe* I	CCGCTAGCCAATCCCTCAAA ACTGT
PR228	Sense oligo for *TbAIR9* fragment (bp 2253-2976)	*Hind* III	GGAAGCTTGAGTTGGAAGTG GAGTAC
PR229	Antisense oligo for *TbAIR9* fragment (bp 2253-2976)	*Xba* I	CGCTCTAGATGCGCCGTCAG TCCG
OL2696	Sense oligo for *TbAIR9* fragment (bp 1803-2215)	*Hind* III	GATGAAGCTTGGATGTGGGC AAGTGTTTG
OL2697	Antisense oligo for *TbAIR9* fragment (bp 1803-2215)	*Bam* HI	GAGGGGATCCGAAGTTCGTC TGGGGAATG
